# GSK3‐ARC/Arg3.1 and GSK3‐Wnt signaling axes trigger amyloid‐β accumulation and neuroinflammation in middle‐aged Shugoshin 1 mice

**DOI:** 10.1111/acel.13221

**Published:** 2020-08-28

**Authors:** Chinthalapally V. Rao, Mudassir Farooqui, Avanish Madhavaram, Yuting Zhang, Adam S. Asch, Hiroshi Y. Yamada

**Affiliations:** ^1^ Hematology/Oncology Section Department of Medicine Center for Cancer Prevention and Drug Development University of Oklahoma Health Sciences Center (OUHSC Oklahoma City Oklahoma USA; ^2^ Department of Neurology University of Iowa Hospitals and Clinics Iowa City Iowa USA; ^3^ Biology/Exercise and Sports Science University of North Carolina Chapel Hill North Carolina USA; ^4^ Hematology/Oncology Section Department of Medicine Stephenson Cancer Center University of Oklahoma Health Sciences Center (OUHSC Oklahoma City Oklahoma USA

**Keywords:** amyloid‐β, cohesinopathy, genomic instability, late‐onset Alzheimer's disease, mitosis, mouse model, neuroinflammation, Shugoshin1

## Abstract

The cerebral amyloid‐β accumulation that begins in middle age is considered the critical triggering event in the pathogenesis of late‐onset Alzheimer's disease (LOAD). However, the molecular mechanism remains elusive. The Shugoshin 1 (Sgo1^−/+^) mouse model, a model for mitotic cohesinopathy‐genomic instability that is observed in human AD at a higher rate, showed spontaneous accumulation of amyloid‐β in the brain at old age. With the model, novel insights into the molecular mechanism of LOAD development are anticipated. In this study, the initial appearance of cerebral amyloid‐β accumulation was determined as 15‐18 months of age (late middle age) in the Sgo1^−/+^ model. The amyloid‐β accumulation was associated with unexpected GSK3α/β inactivation, Wnt signaling activation, and ARC/Arg3.1 accumulation, suggesting involvement of both the GSK3‐Arc/Arg3.1 axis and the GSK3‐Wnt axis. As observed in human AD brains, neuroinflammation with IFN‐γ expression occurred with amyloid‐β accumulation and was pronounced in the aged (24‐month‐old) Sgo1^−/+^ model mice. AD‐relevant protein panels (oxidative stress defense, mitochondrial energy metabolism, and β‐oxidation and peroxisome) analysis indicated (a) early increases in Pdk1 and Phb in middle‐aged Sgo1^−/+^ brains, and (b) misregulations in 32 proteins among 130 proteins tested in old age. Thus, initial amyloid‐β accumulation in the Sgo1^−/+^ model is suggested to be triggered by GSK3 inactivation and the resulting Wnt activation and ARC/Arg3.1 accumulation. The model displayed characteristics and affected pathways similar to those of human LOAD including neuroinflammation, demonstrating its potential as a study tool for the LOAD development mechanism and for preclinical AD drug research and development.

## Graphical abstract

In late middle age of a genomic instability mouse model (Sgo1^−/+^), unexpected inactivation of GSK3 leads to amyloid‐beta accumulation in the brain via two signaling axes: GSK3‐ARC/Arg3.1 axis and canonical Wnt/GSK3‐beta‐catenin axis. This study cautions GSK3 inhibition in the middle age for possibly triggering late‐onset Alzheimer's disease.

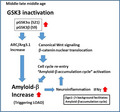

**Abbreviations**
Aco2: Aconitase 2, mitochondrial
AD: Alzheimer's disease
APOE: Apolipoprotein E
APP: Amyloid Precursor Protein
ARC/Arg3.1: Activity‐regulated cytoskeleton‐associated protein
BACE1: β‐secretase 1
COX‐2: cyclooxygenase 2
EBF3: Early B‐cell Factor 3
EOAD: early‐onset Alzheimer's disease
Fabp3: fatty acid‐binding protein 3
Fh1: Fumarate Hydratase
GFAP: Glial fibrillary acidic protein
Gpi: Glucose‐6‐phosphate isomerase
GSK3: Glycogen synthase kinase 3
Hspd1: Heat Shock Protein Family D [Hsp60] Member 1
Idh1: Isocitrate Dehydrogenase [NADP(+)] 1, Cytosolic
Idh2: Isocitrate Dehydrogenase [NADP(+)] 2, Mitochondrial
IFN‐γ: Interferon‐γ
IL10: Interleukin 10
IL1‐β: Interleukin1‐β
LOAD: late‐onset Alzheimer's disease
mtUPR: mitochondrial unfolded protein response
NDUFV1: NADH:Ubiquinone Oxidoreductase Core Subunit V1
NFT: Neurofibrillary Tangles
NFκB65: Nuclear Factor‐κB 65kd subunit
nnt: Nicotinamide Nucleotide Transhydrogenase
Pdk1: Pyruvate Dehydrogenase Kinase 1
PGE2: Prostaglandin E2
Phb: Prohibitin
Phb2: Prohibitin2
PKM2: Pyruvate kinase isozyme M2
p‐MAPK: phosphor‐mitogen activated protein kinase
prdx6: Perredoxin6
PSEN1: Presenilin 1
Sgo1: Shugoshin 1
TNF‐α: Tumor necrosis factor‐α

## 1 INTRODUCTION

### 1.1 Unmet need for AD drug development

With an increasing number of patients and no effective treatments, AD drug represents a major unmet need in medicine. As of early 2020, more than 200 clinical trials for AD have failed, including 9 phase 3 trials since 2016 (Cummings et al., 2016; Cummings, Morstorf, & Zhong, 2014; Huang, Chao, & Hu, 2020). The high failure rate may be attributed to (a) incomplete understanding of human AD pathology development, and (b) insufficient representation of late‐onset Alzheimer's disease (LOAD) models in the AD drug research and development (R&D) process.

### 1.2 Hypotheses for load pathology development

Drug development is driven by hypotheses. Strong hypotheses that are well‐supported by data are needed for clinical AD drug development. A number of hypotheses have been proposed for human AD development and its cause, including the “neuroinflammation hypothesis,” the “cholinergic hypothesis,” the “Tau hypothesis,” the “infection hypothesis,” and the currently prevailing “amyloid‐β oligomer hypothesis” (Fan et al., 2020). However, which hypothesis will lead to effective clinical therapeutics remains to be seen. Some hypotheses lost support or have received less enthusiasm, due to limited efficacy with or side effects of target‐specific drugs (e.g., acetylcholinesterase inhibitors and NSAIDs).

### 1.3 Limited modeling for load

LOAD accounts for over 95% of all human AD. The remaining 2%–5% of cases are early‐onset AD (EOAD) that are linked to genetic mutation(s) in genes involved in amyloid metabolism, such as Amyloid Precursor Protein (APP), Presenilin 1 (PSEN1), and Presenilin 2 (PSEN2). With insufficient knowledge in modeling LOAD, most preclinical AD drug R&D has employed rodent EOAD models. However, most LOAD patients do not carry mutation(s) in EOAD genes (Carmona, Hardy, & Guerreiro, 2018; Giri, Zhang, & Lü, 2016), suggesting other, and likely multiple, causes influencing LOAD pathology, such as APOE‐associated transports. Many AD drug candidates that showed efficacy in EOAD mice have failed in clinical trials. These findings led to uncertainty concerning EOAD models for proper target representation in human AD drug R&D. For example, although many of the existing EOAD mice models display signs of microgliosis and astrogliosis with development of amyloid‐β deposits, these mice generally do not show robust neuroinflammation comparable to that in human AD patients (Drummond & Wisniewski, 2017; Jankowsky & Zheng, 2017; Saito & Saido, 2018; Sasaguri et al., 2017). This uncertainty calls for a research model that does not rely on EOAD mutations, while representing some aspects of LOAD (i.e., LOAD model).

### 1.4 Shugoshin 1 (Sgo1)^−/+^ mouse model

In 2018, a mouse model that spontaneously accumulates amyloid‐β in the brain at old age was reported (Rao, Farooqui, Zhang, Asch, & Yamada, 2018). The model, the Shugoshin1 haploinsufficient mouse (Sgo1^−/+^), was originally developed for carcinogenesis studies, as genomic instability and aneuploidy had long been hypothesized to cause cancer (Holland & Cleveland, 2009). Genomic instability mouse models with a focus on carcinogenesis, including the Sgo1^−/+^ mouse, have revealed dual oncogenic and tumor‐suppressive effects of genomic instability on cancer (Rao, Asch, & Yamada, 2017; Schvartzman, Sotillo, & Benezra, 2010; Simon, Bakker, & Foijer, 2015) and aided advancing understanding of tumor evolution (Sansregret & Swanton, 2017). However, other biological events associated with genomic instability (e.g., aging and Alzheimer's disease) had not been explored. Among genomic instability models, the Sgo1^−/+^ model is designed to increase the rate of mitotic cohesinopathy, a genomic instability‐inducing event that is also naturally occurring in human aging oocytes and frequently seen in peripheral blood, buccal cells, and neurons of patients with AD (Andriani, Vijg, & Montagna, 2017; Bajic, Spremo‐Potparevic, Zivkovic, Isenovic, & Arendt, 2015; Hou, Song, Croteau, Akbari, & Bohr, 2017; Lee, Thomas, & Fenech, 2015; Shepherd, Yang, & Halliday, 2018; Zivković et al., 2010). As such, we hypothesized that Sgo1^−/+^ causes AD pathology (Rao, Farooqui, Zhang, et al., 2018). The Sgo1 protein functions as a protective glue between mitotic chromosomes and in centrosome, maintaining the structural integrity of cohesion between mitotic chromosomes and of centrosomes (Salic, Waters, & Mitchison, 2004; Schöckel, Möckel, Mayer, Boos, & Stemmann, 2011; Yamada et al., 2012). The Sgo1^−/+^ mouse brains differentially expressed genes associated with neuronal functions and human AD, including Activity‐regulated cytoskeleton‐associated protein (ARC/Arg3.1) and Early B‐cell Factor 3 (EBF3) in old age (24 months) (Rao, Farooqui, Zhang, et al., 2018). Hence, the model is a candidate for the first genetically‐defined mouse model for LOAD, once it is fully validated with cognitive function analysis and other molecular analysis. The International Mouse Phenotyping Consortium (IMPC) database reports an abnormal behavior phenotype in Sgo1^tm1a(EUCOMM)Wtsi^ allele mice, suggesting the likelihood of AD‐like cognitive function/behavioral issues with Sgo1 defects (The IMPC database website: http://www.mousephenotype.org/data/genes/MGI:1919665#section‐associations). Previously, spontaneous accumulation of cerebral amyloid‐β was not thought to occur in mice, and it was attributed to the protein sequence difference and shorter lifespan (Drummond & Wisniewski, 2017; Sasaguri et al., 2017). The Sgo1^−/+^ mouse provided a novel observation counter to the previously prevailing notion. There is the potential for the Sgo1^−/+^ mitotic cohesion defect‐genomic instability model to serve as a LOAD model portraying aspects of the disease that were not represented in previous models.

### 1.5 A novel integrated hypothesis for amyloid‐β accumulation; the “amyloid‐β accumulation cycle”

Amyloid‐β accumulation in the Sgo1^−/+^ model was originally hypothesized to be linked to three events: (a) aging, (b) mitotic re‐entry, and (c) prolonged mitosis [the “Three‐hit” hypothesis] (Rao, Farooqui, Asch, & Yamada, 2018). Recently, incorporating evidence suggestive of mitotic dysregulations as a common underlying, if not causal, event for both early‐onset and late‐onset human AD, an integrated hypothesis for amyloid‐β accumulation, the “amyloid‐β accumulation cycle,” was proposed (Rao, Asch, Carr, & Yamada, 2020). The “amyloid‐β accumulation cycle” hypothesis purports the occurrence of vicious cycles of events leading to amyloid‐β accumulation, as follows: initial increase in amyloid‐β, growth signaling activation and inflammation triggered by the amyloid‐β exposure, mitotic re‐entry, accumulation of amyloid‐β during (quasi‐)mitotic state, mitotic catastrophe, and release of more amyloid‐β from dead cells into microenvironment, leading to another cycle. The cycle is enabled by unique characteristics of amyloid‐β. Amyloid‐β is neurotoxic, can activate growth signaling, is pro‐inflammatory, can interfere with mitosis (aneuploidogenic), and can be generated during mitosis or in quasi‐mitosis conditions. The cycle may occur at any time during LOAD development, in early and in late stages. Yet, amyloid‐β catabolism, which can antagonize the cycle and may decline over age, may be a factor influencing the late‐onset accumulation of amyloid‐β (Rao et al., 2020).

### 1.6 Purpose of this study

The observation of amyloid‐β accumulation in the aged Sgo1^−/+^ model mice raised two major questions; (a) whether the mouse model reflects or recapitulates some other aspects of human LOAD pathology and its development process, such as neuroinflammation and oxidative stress, and (b) in light of the “amyloid‐β accumulation cycle” hypothesis, which growth signaling is/are responsible for the amyloid‐β accumulation in the model. In the present study, we (a) determined the timing of amyloid‐β appearance over age, (b) tested a hypothesis that human AD‐relevant age‐associated events (i.e., neuroinflammation, misregulations in proteins involved in oxidative stress, mitochondrial energy metabolism, or β‐oxidation/peroxisome) are associated with amyloid‐β accumulation in the Sgo1^−/+^ model, and (c) identified the GSK3‐ARC/Arg3.1 and GSK3‐Wnt signaling axes as candidates for triggering amyloid‐β accumulation in middle age.

## 2 METHODS

### 2.1 Samples

Twenty‐four‐month‐old animals and brain tissue samples were obtained as described in Rao, Farooqui, Zhang, et al. (2018). Twelve‐month‐old animals and brain tissue samples were obtained from a previous study described in Yamada et al., (2015). Fifteen‐ and eighteen‐month‐old Sgo1^−/+^ brain tissue samples were obtained in the present study (*N* = 4‐5) from both genders (2‐5 each). We have not observed significant differences in male/female birth ratio, cancer or other disease development, and Aβ accumulation, between genders in Sgo1^−/+^ mice. At sample collection, whole brains were cut in half at the midline. Left hemispheres were fixed in 10% buffered formalin, then were embedded in paraffin and processed onto slides in the CCPDD histopathology core for immunohistochemistry or immunofluorescence. Right hemispheres were flash‐frozen in liquid nitrogen and stored at −80°C. For immunoblots and select pathway protein panel analyses, the rear parts of forebrains, including cortex and hippocampus, were excised and used.

### 2.2 Immunoblots, immunohistochemistry, and immunofluorescence

For immunoblots and immunofluorescence, the procedures described in Rao, Farooqui, Zhang, et al. (2018) were followed. For immunoblot control, rat/mouse amyloid‐β1‐42 synthetic peptide (Abcam; ab120959) was used. Since formalin‐fixed aged brain samples tend to generate high autofluorescence, sodium borohydride and CuSO_4_ treatments were included as our standard procedures for immunofluorescence. For immunohistochemistry, we used the SuperPicture 3rd gen IHC kit (Thermo Fisher Scientific) following the manufacturer's protocol. We used the following primary antibodies: Actin (Santa Cruz Biotechnology; Cat. number, sc‐1616), amyloid‐β (referred as B‐4; Santa Cruz, sc‐28365), amyloid‐β (referred as NAB228; Cell Signaling Technology; #2450), amyloid‐β (referred as D54D2; Cell Signaling Technology, #8243), COX‐2 (Santa Cruz, sc‐7951), GFAP (Thermo Fischer Scientific, Rb‐087‐A), GSK3α+β (Cell Signaling Technology, # 5676T), Iba1 (Abcam, ab178846), IFN‐γ (Bioss antibodies, bs‐0480R), IL1‐β (Bioss, bs‐6319R), IL‐6 (Bioss, bs‐0379R), IL10 (Bioss, bs‐0698R), NFκB 65kd (Santa Cruz, sc‐372), phosphor‐p38MAPK [T180+Y182] (Bioss, bs‐2210R), p21^WAF1/CIP1^ (Santa Cruz, sc‐817), p‐GSK3α (S21) (Cell Signaling Technology, #9316), p‐GSK3β (S9) (Biorbyt, # orb393067), p‐TAU(S262) (EnoGene; E011111), p‐TAU (S404) (Santa Cruz, sc‐12952), and TNF‐α (Bioss, bs‐2081R).

### 2.3 Pathway protein panel analysis

Frozen mouse brains (mouse cerebrum, including cortex and hippocampus, and excluding olfactory bulb, cerebellum, and medulla) were extracted in RIPA buffer with 250 mM NaCl, with added protease inhibitor cocktail (Sigma‐Aldrich) and proteasome inhibitor MG132 10 µM (Sigma‐Aldrich). Extracts were cleared with 5000 rpm for 5 min. Protein concentrations of the supernatants were estimated with a Nanodrop spectrophotometer (Thermo Fisher Scientific).

The supernatants were submitted to the Multiplexing Protein Quantification Core facility at the Oklahoma Medical Research Foundation (Oklahoma City, OK, USA) for protein panel analyses with quantitative mass spectrometry (TSQ Quantiva triple quadrupole mass spectrometry system) (https://aging.ouhsc.edu/Cores/Multiplexing‐Protein‐Quantification‐Core).

### 2.4 Quantitative mass spectrometry

The samples were mixed with 100 µl 1% SDS, 20 µl of our Bovine Serum Albumin (BSA) internal standard, mixed, and heated for 15 min. The proteins precipitated with 1 ml acetone. The dried protein pellet was reconstituted in 60 µl Laemmli sample buffer and 20 µl (20 µg) was used to run a short (1.5‐cm) SDS‐PAGE gel. The gels were fixed and stained. Each sample was cut from the gel as the entire lane and divided into smaller pieces. The gel pieces were washed to remove the Coomassie blue, then reduced, alkylated, and digested overnight with trypsin. The mixture of peptides was extracted from the gel, evaporated to dryness in a SpeedVac, and reconstituted in 200 µl 1% acetic acid for analysis.

The analyses were carried out on a TSQ Quantiva triple quadrupole mass spectrometry system. The HPLC was conducted on an Ultimate 3000 nanoflow system with a 10 cm × 75 µm i.d. C18 reversed‐phase capillary column. 5‐µl aliquots were injected and the peptide was eluted with a 60‐min gradient of acetonitrile in 0.1% formic acid.

The mass spectrometer was operated in the selected reaction monitoring mode. For each protein, the method was developed to measure two ideal peptides. Assays for multiple proteins were bundled together in larger panels. Data were analyzed using the program Skyline to determine the integrated peak area of the appropriate chromatographic peaks. The response for each protein was calculated as the geometric mean of the peptide areas. These values were normalized to the response for the BSA standard and to the total ion current. The samples were also analyzed on our Thermo QEx system in the LC‐full scan MS mode. The total ion current in those analyses is an indication of the amount of material present in the sample and may be useful for normalization. For final analysis, we used total ion current for normalization, as we found some variations among BSA signals. Additional “universal detection” runs, high‐resolution accurate mass (HRAM), were also done using our orbitrap system (Thermo Scientific QEx plus), as an additional type of data that can be re‐interrogated as needed.

Overall, as above, four groups (12‐month‐old wild‐type, 12‐month‐old Sgo1^−/+^, 24‐month‐old wild‐type, and 24‐month‐old Sgo1^−/+^; *N* = 5 each) with a total of 20 samples were simultaneously processed for quantification. The amounts of proteins (i.e., representative peptides) in the panels were quantified. Total ion current was used for normalization. An antioxidant protein panel (49 proteins), a mitochondria and energy metabolism panel (47 proteins), and a β‐oxidation and peroxisome panel (37 proteins) were analyzed. The panels were 2018 July/August version. Subtracting overlapping proteins in the panels, a total of 130 proteins were analyzed (see Figure S1).

### 2.5 Cohort comparison

To identify differences among the four groups, normalized peptide reads were analyzed with a series of group‐to‐group comparisons; (a) 12‐month‐old wild‐type vs. 12‐month‐old Sgo1^−/+^, (b) 24‐month‐old wild‐type vs. 24‐month‐old Sgo1^−/+^, (c) 12‐month‐old wild‐type vs. 24‐month‐old wild‐type, and (d) 12‐month‐old Sgo1^−/+^ vs. 24‐month‐old Sgo1^−/+^, with GraphPad Prism 7 software (ver7.03). Unpaired *t*‐test results with *N* = 5, *p* < 0.05, calculated by the software, were considered significant. Simultaneous multiple comparisons (e.g., two‐way ANOVA) were not employed, as the appropriate correction method factoring both age‐associated effects and strain‐associated effects has not been determined.

## 3 RESULTS

### 3.1 Validation of antibodies

**FIGURE 1:**
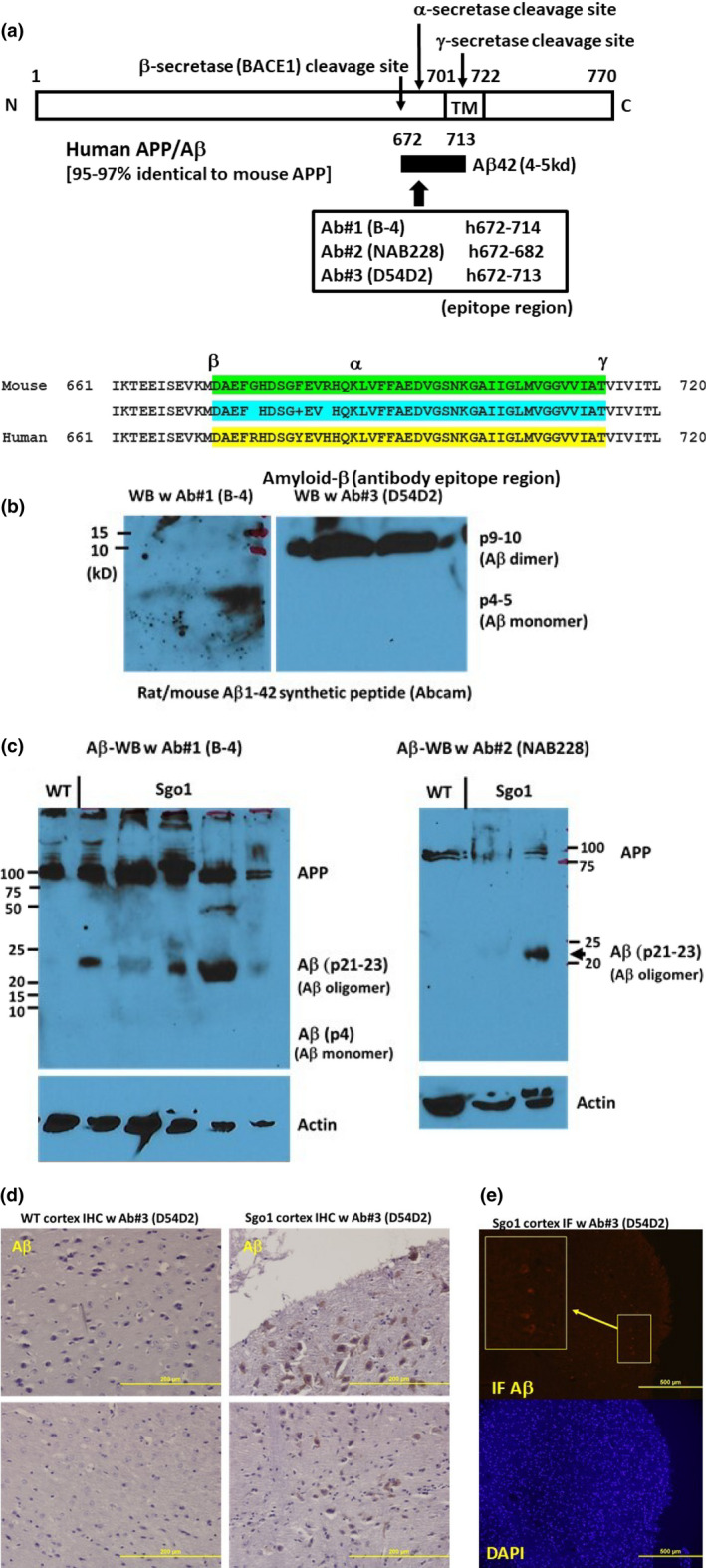
Cerebral amyloid‐β is accumulated in aged (24 month‐old) Sgo1^−/+^ mice. (a) Human/mouse APP structure. Anti‐amyloid‐β antibodies used for this study were generated against human amyloid‐β, which is 97% identical to mouse amyloid‐β. In humans, B‐4 and NAB228 recognize both APP and amyloid‐β, while D54D2 preferentially recognizes amyloid‐β. (b) Synthetic rat/mouse amyloid‐β^1−42^ peptide was recognized by anti‐ amyloid‐β antibodies. Amyloid‐β can form SDS‐resistant oligomers that may expose epitope regions differently. B‐4 antibody preferentially recognized monomer (p4‐5), while D54D2 recognized dimer (p9‐10). (c) Twenty‐four‐month‐old Sgo1^−/+^ brain extracts showed amyloid‐β p21‐23. Both anti‐amyloid‐β antibodies, B‐4 (left panel) and NAB228 (right panel), detected APP and amyloid‐β p21‐23 in immunoblots. Immunoblots of extracts from age‐matched wild‐type mice detected only APP. (d) Twenty‐four ‐month‐old Sgo1^−/+^ brain showed amyloid‐β accumulation in IHC. Control age‐matched wild‐type mice did not show IHC‐positive staining. (e) Twenty‐four‐month‐old Sgo1^−/+^ brain showed amyloid‐β accumulation in IF. IF showed positive signals in Sgo1^−/+^ brain, consistent with IHC results. Enlarged panel shows the signals from cell bodies. Control wild‐type mice did not show clear signals with equalized image acquisition settings (not shown)

Mouse APP is 95%–97% identical to human APP (Figure 1a). To verify amyloid‐β accumulation in mice, we used three commercial antibodies raised for human amyloid‐β (APP/amyloid‐β‐recognizing B‐4 and NAB228, and amyloid‐β‐specific D54D2), with synthetic rat/mouse amyloid‐β peptide as a control. This series of pilot experiments verified that these antibodies recognize mouse amyloid‐β. In immunoblots, amyloid‐β can form SDS‐resistant oligomers and can appear in monomer (p4‐5), dimer (p9‐10), and higher molecular weight oligomers (e.g., p21‐23 and p35). B‐4 antibody preferentially recognized the p4‐5 monomer form of mouse synthetic amyloid‐β, while D54D2 recognized the p9‐10 dimer form (Figure 1b). In addition to oligomerization, *in vivo* APP can be cleaved by a variety of proteases (e.g., α‐,β‐,γ‐δ‐,η‐secretases, and Neprilysin/CD10), which can lead to generation of protein fragments of different sizes (Andrew, Kellett, Thinakaran, & Hooper, 2016; Wilkins & Swerdlow, 2017). We tested 24‐month‐old Sgo1^−/+^ brain extracts with the antibodies. Control age‐matched wild‐type extracts showed only full‐length APP (p87) and no shorter form, while Sgo1^−/+^ brain extracts showed p21‐23 (and p50 in some mice) in addition to APP. Both B‐4 and NAB228 antibodies indicated the same‐sized band of p21‐23, which we interpret as an oligomer form of mouse amyloid‐β (Figure 1c). In 18‐month‐old PS1×APP EOAD mice, in which human transgenes for both APP‐SWE and PSEN1‐L166P mutations are expressed under the Thy1 promoter, soluble SDS‐resistant Aβ oligomeric species of approximately 24, 50, 60, and 90 kDa were reported, and p24 (corresponding to our p21‐23) was estimated as 6‐mer of Aβ (Jimenez et al., 2011). We also tested localization of the mouse amyloid‐β with immunohistochemistry (IHC) (Figure 1d) and immunofluorescence (IF) (Figure 1e). As in immunoblots, wild‐type control brains did not show IHC‐positive signals, while Sgo1^−/+^ brains showed IHC‐positive signals in cell bodies (Figure 1d). Results of IF with equalized acquisition settings were consistent with IHC findings; no signal was detected in wild‐type brains, while IF‐positive cells were observed in Sgo1^−/+^ brains (Figure 1e). The same D54D2 antibody showed IHC signals in HSV1‐infected wild‐type mice (Rao et al., 2020), a condition in which mouse amyloid‐β was also accumulated (De Chiara et al., 2019). Overall, the results indicate that mouse amyloid‐β accumulates in aged Sgo1^−/+^ mouse brains.

### 3.2 Cerebral amyloid‐β started accumulating at late middle age (15‐18 months of age)

**FIGURE 2:**
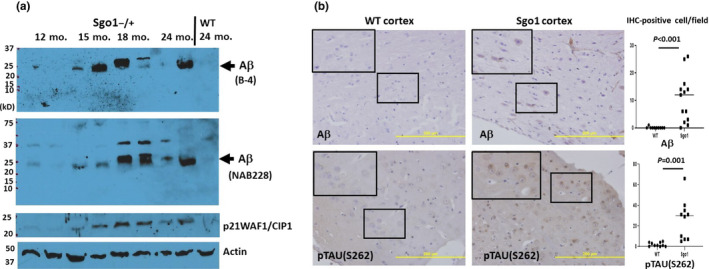
Late‐onset nature of cerebral amyloid‐β accumulation in Sgo1^−/+^ mice. (a) Cerebral amyloid‐β accumulated by 18 months of age in Sgo1^−/+^ mice. Extracts were prepared from brains from Sgo1^−/+^ mice at 12, 15, 18, and 24 months of age. Extracts were probed for amyloid‐β (with B‐4, top panel), amyloid‐β (with NAB228, middle panel), aging marker p21^WAF1/CIP1^, and actin (loading control). (b) amyloid‐β accumulation in Sgo1^−/+^ brain cortex occurred in cell bodies. Amyloid‐β IHC with D54D2 antibody for 18‐month‐old Sgo1^−/+^ brain showed positive signals. However, even at 24 months of age, wild‐type mice showed no positive signals. Bar = 200 µm. Marked fields are enlarged to show IHC details; Aβ and p‐TAU (S262) were observed in cell bodies in Sgo1^−/+^ cortex, while no signal was seen in wild‐type. IHC‐positive cells were counted in pictures of cortex (*N* = 10–13), and the number of IHC‐positive cells per field is presented. (c) p‐TAU^S262^ staining appeared in 18‐month‐old Sgo1^−/+^ brain. As with amyloid‐β, even at 24 months of age, wild‐type mice showed no positive staining

Next, we investigated the timing of amyloid‐β accumulation in Sgo1^−/+^ mice. In previous analysis, 12‐month‐old Sgo1^−/+^ mice did not show cerebral amyloid‐β accumulation, while 24‐month‐old Sgo1^−/+^ mice showed cerebral amyloid‐β accumulation (Rao, Farooqui, Asch, et al., 2018; Rao, Farooqui, Zhang, et al., 2018). We tested brains from Sgo1^−/+^ mice of different ages (12, 15, 18, and 24 months of age) with immunoblots and IHC (Figure 2a,b). Immunoblots indicated that amyloid‐β p21‐23 in Sgo1^−/+^ mice initially appeared around 15 months of age, and manifested by 18 months of age (late middle age). IHC showed that amyloid‐β accumulated mainly in the cytoplasm of cell bodies in the cortex, but not as extracellular plaques at this age. Control wild‐type mice did not show signs of amyloid‐β, even at 24 months of age. Expression of another major AD marker, p‐TAU, was weak if present, when tested with immunoblots with pTAU S404 and S262 antibodies. However, localized accumulation of pTAU^S262^ in cell bodies was observed with IHC (Figure 2b). Expression of aging biomarker p21^WAF1/CIP1^, the variants of which are also a risk factor for human AD (Yates et al., 2015), prematurely increased in 15‐to‐18‐month‐old Sgo1^−/+^ mice. The p21 increase was concurrent with Aβ increase. Expression of p21^WAF1/CIP1^ remained low in wild‐type mice even at 24 months of age. Thus, cerebral amyloid‐β accumulation in Sgo1^−/+^ mice is late‐onset occurring past middle age, progressive with age, and concurrent with p21^WAF1/CIP1^ increase.

### 3.3 INF‐γ‐mediated neuroinflammation in aged Sgo1^−/+^

**FIGURE 3:**
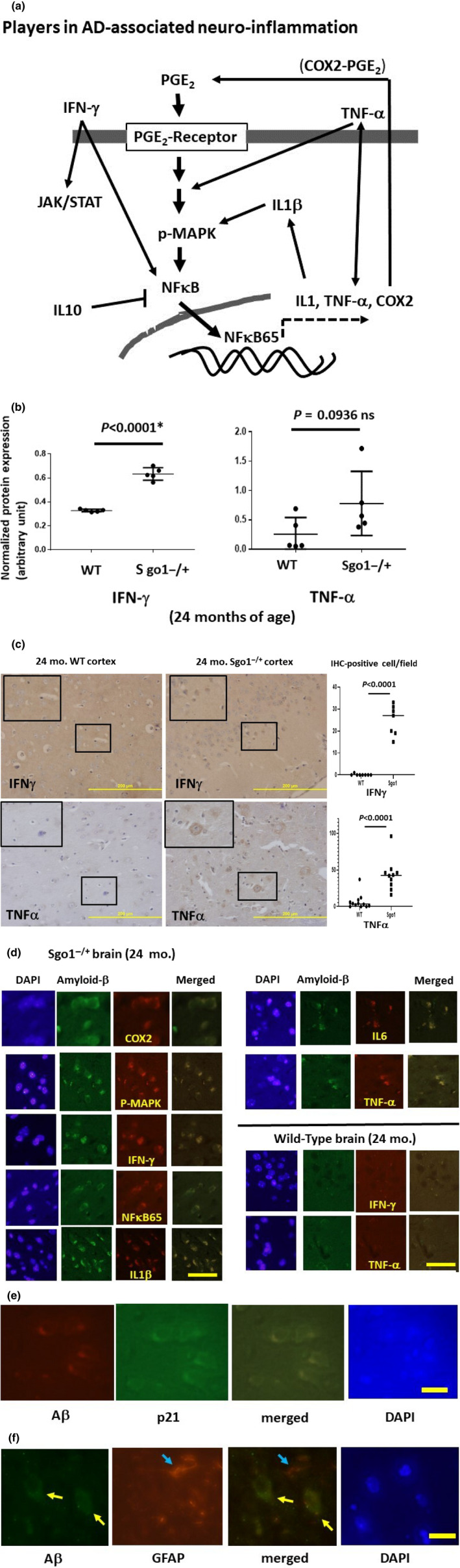
Neuroinflammation in Sgo1^−/+^ mice. (a) Players in AD‐associated neuroinflammation. For this analysis, we prioritized neuroinflammation markers that are known to be upregulated (i.e., IFN‐γ, TNF‐α, NFκB65, IL1‐β, IL6, and COX‐2) or downregulated (i.e., IL10) in the brains of human patients with AD. Overall, the neuroinflammation markers are proposed to form feedback loops. One messenger is Prostaglandin E_2_ (PGE_2_), which binds to the receptor and activates a cascade, leading to MAPK phosphorylation and activation of the NFκB 65kd subunit. The NFκB 65kd subunit translocates to the nucleus and activates downstream transcriptions of other inflammatory mediators, including IL1, TNF‐α, and COX‐2. COX‐2, in turn, generates PGE_2_. Inflammatory cytokine IL1‐β activates COX‐2. TNF‐α is also thought to activate p38MAPK. Inflammatory cytokine Interferon‐γ (IFN‐γ) leads to activations of JAK/STAT and NFκB. However, interpreting the role of neuroinflammation markers may not be straightforward. (b) AD‐associated neuroinflammation marker IFN‐γ was upregulated in aged, amyloid‐β‐accumulating Sgo1^−/+^ brains (*p* < 0.0001). Another marker, TNF‐α, was consistently upregulated in Sgo1^−/+^, showing the dual presence of IFN‐γ and TNF‐α in Sgo1^−/+^ brain. However, inconsistent expression of TNF‐α in wild‐type control mice led to a non‐significant *p*‐value (*p* = .0936). (c) IFN‐γ and TNF‐α in Sgo1^−/+^ brain. As suggested by immunoblot results in (b), IFN‐γ and TNF‐α were both detected in aged Sgo1^−/+^ brain. Also consistent with immunoblots, IHC of wild‐type controls showed much less IFN‐γ and TNF‐α. IFN‐γ and TNF‐α were observed in cell bodies in Sgo1^−/+^ cortex, while no signal was seen in wild‐type (enlarged panels). IHC‐positive cells were counted in pictures of cortex (*N* = 7–12), and the number of IHC‐positive cells per field is presented. (d) Accumulating amyloid‐β and neuroinflammation markers (COX2, p‐MAPK, IFN‐γ, NFκB65, IL1‐β, IL6, and TNF‐α) co‐localized in Sgo1^−/+^ brain cortex cells. Age‐matched wild‐type mice showed little amyloid‐β, and co‐localization was not observed. Scale bar (yellow):50 µm. (e) Amyloid‐β and p21 co‐localized in Sgo1^−/+^ brain cortex cells. Scale bar (yellow):20 µm. (f) Amyloid‐β and Glial fibrillary acidic protein (GFAP) did not co‐localize in Sgo1^−/+^ brain cortex cells. GFAP is a marker for astrocytes and ependymal cells. Aβ‐positive cells (yellow arrow) and GFAP‐positive cells (blue arrow) are different cells. Scale bar (yellow): 20 µm

Various neuroinflammation markers, including NFκB, IL1‐β, IFN‐γ, TNF‐α, and p‐MAPK, are misregulated in the brains of patients with AD (see Figure 3a) (Fan et al., 2020; Saito & Saido, 2018; Skaper, Facci, Zusso, & Giusti, 2018). Bredesen's group proposed that human AD can be categorized into three subtypes; inflammatory, non‐inflammatory, and atypical (Bredesen, 2015, 2017). We tested whether Sgo1^−/+^‐mediated amyloid‐β accumulation is associated with neuroinflammation.

First, we compared total amounts of IFN‐γ and TNF‐α, as well as IL10, NFκB65, and IL1‐β. Twenty‐four‐month‐old Sgo1^−/+^ mice showed consistent expression of IFN‐γ, while IFN‐γ was undetectable in age‐matched wild‐type mice (*p* < 0.0001) (Figure 3b). TNF‐α expressions were noted in all Sgo1^−/+^ mice, indicating co‐expressions of IFN‐γ and TNF‐α in the Sgo1^−/+^ model. However, inconsistent expression in wild‐type mice resulted in a lack of statistical significance for TNF‐α overexpression (*p* = .093; Figure 3b). Consistently, cell body staining for IFN‐γ and TNF‐α in the aged Sgo1^−/+^ model mice was observed in IHC, while little IHC staining was observed for wild‐type mice (Figure 3c). There were no significant differences in total amounts of NFκB65kd, IL10, and IL1‐β in control and Sgo1^−/+^ mice (not shown). However, co‐localization between amyloid‐β and COX2, p‐MAPK, IFN‐γ, NFκB65kd, IL1‐β, IL6, and TNF‐α were observed in Sgo1^−/+^ mice (Figure 3d), while co‐localization between amyloid‐β and p16 was not (not shown). Wild‐type mice expressed little to no amyloid‐β, and no notable co‐localizations in these markers were observed in these mice (Figure 3d). The results indicated that neuroinflammation occurs in aged Sgo1^−/+^ mice that accumulated amyloid‐β. We additionally tested whether p21 also co‐localizes with amyloid‐β (Figure 3e). Amyloid‐β signals co‐localized with p21, corroborating with immunoblots in Figure 2. However, astrocyte/ependymal cell marker GFAP did not co‐localize with amyloid‐β (Figure 3f). The exact cell type(s) accumulating amyloid‐β is under investigation.

### 3.4 The middle‐aged Aβ accumulation is concurrent with IFN‐γ‐mediated neuroinflammation

**FIGURE 4:**
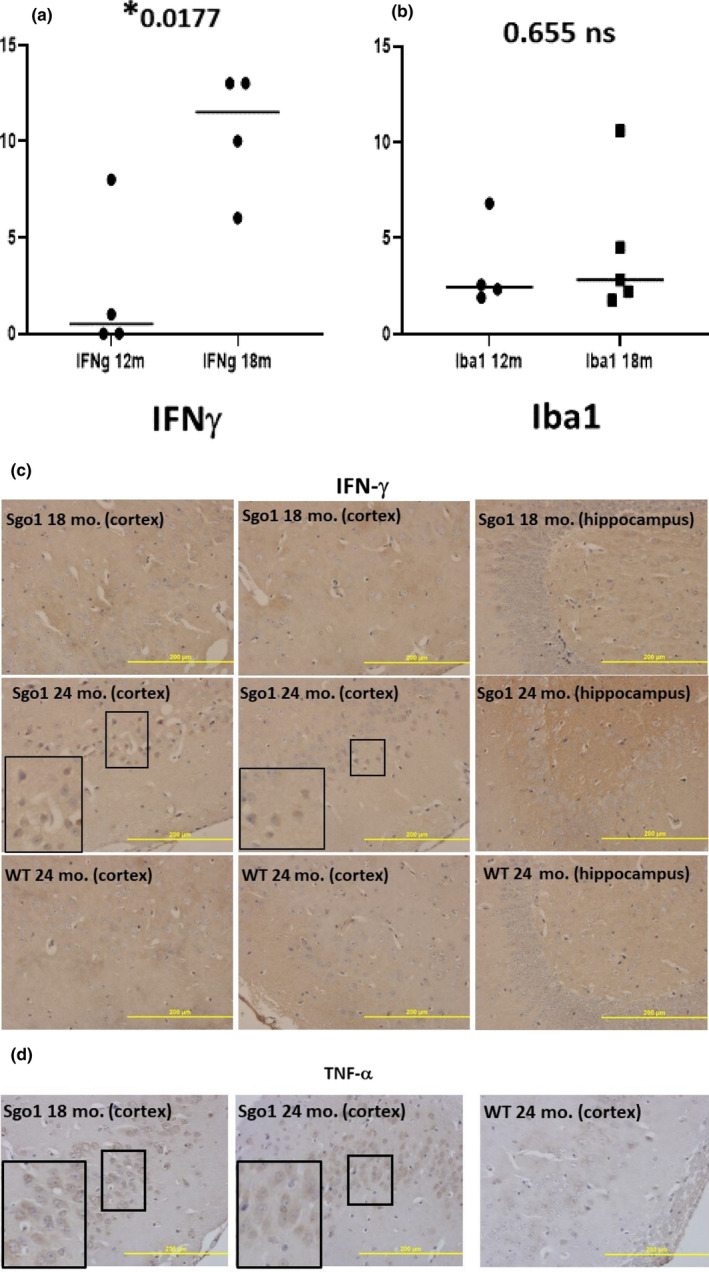
IFN‐γ ‐mediated neuroinflammation was concurrent with, but did not precede, Aβ accumulation. (a) IFN‐γ increased by age 18 months in Sgo1^−/+^. The amount of IFN‐γ in Sgo1^−/+^ brain was low at 12 months of age, but increased by 18 months of age. Protein amounts, measured by immunoblot and normalized with actin amount, are plotted and compared. (b) Microglia infiltration may not accompany IFN‐γ increase at the 12‐18 month transition. Microglia marker Iba1 (measured as in (a)) did not show a significant increase in 18‐month‐old Sgo1^−/+^, suggesting that infiltration of microglia may not be significantly increased at this age. (c) IFN‐γ localized in nucleo‐cytoplasm in aged Sgo1^−/+^. Distinct localization of IFN‐γ in nucleo‐cytoplasm was observed only in aged (24‐month) samples and was not observed in 18‐month samples. Marked fields are enlarged to show IHC details. IFN‐γ was observed in cell bodies in 24‐month Sgo1^−/+^ cortex, while no localized signal was seen in 18‐month Sgo1^−/+^ brain samples. Wild‐type controls did not show IFN‐γ at any age. (d) TNF‐α localization in cytoplasm and in nucleo‐cytoplasm. Distinct cell body localization of TNF‐α was observed in Sgo1^−/+^ cortex at both 18 and 24 months of age. At 18 months, cytoplasmic staining was evident (enlarged panel), while at 24 months, diffused staining in both nucleoplasm and cytoplasm was common

Neuroinflammation is proposed to be a major disease modifier for AD. Yet, its causal role in AD development remains controversial. We questioned whether IFN‐γ expression‐mediated neuroinflammation is a triggering event for Aβ accumulation, and investigated the timing when IFN‐γ expression became prominent. The amount of IFN‐γ in Sgo1^−/+^ brain was low at 12 months of age and increased over time (Figure 4a). We next investigated whether the increase of IFN‐γ at 18 months of age is accompanied by activation of microglia. However, clear signs of microglia congressing around Aβ‐positive cells were not obtained (not shown). Iba1, a microglia marker, did not indicate a significant increase (Figure 4b). In 18‐month samples, only diffused IFN‐γ was seen, and distinct localization was observed only in aged 24‐month samples (Figure 4c). Wild‐type controls did not show IFN‐γ at any age (Figure 4c). Distinct cell body localization of TNF‐α was observed in Sgo1^−/+^ cortex at both 18 and 24 months of age (Figure 4d). The results indicate that IFN‐γ ‐mediated neuroinflammation was concurrent with, but did not precede, Aβ accumulation. The results also suggest that activation of microglia at the early phase of Aβ accumulation is weak, if any.

### 3.5 WNT and GSK3α/β signaling were misregulated at the middle age/late middle age transition in Sgo1^−/+^ mice

**FIGURE 5:**
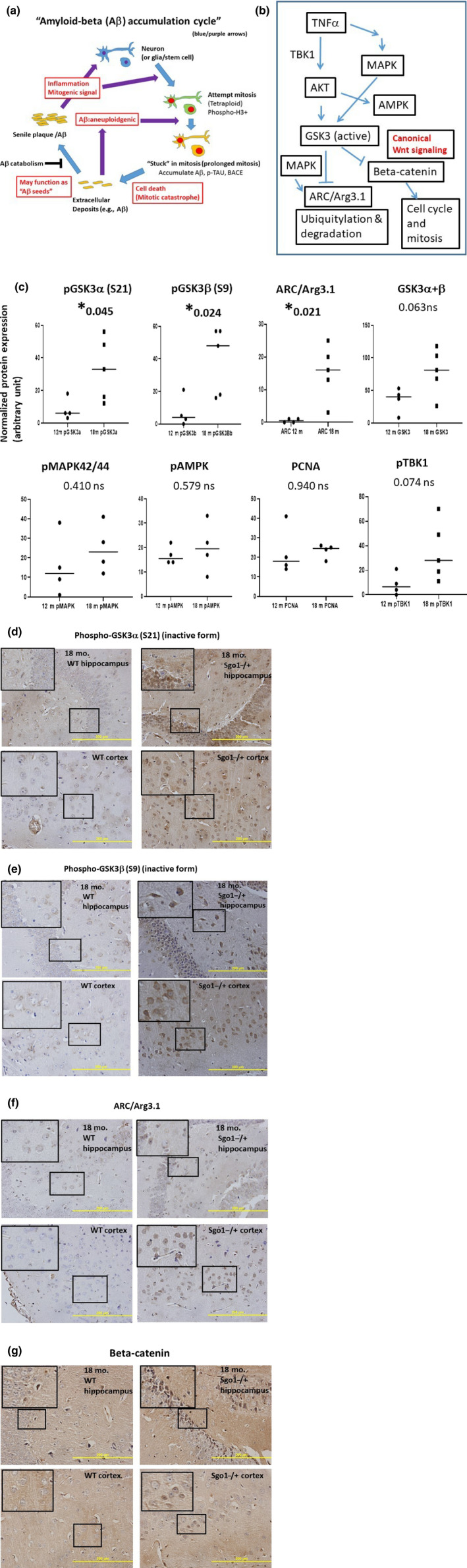
Inhibition of GSK3 α and β, accumulation of ARC/Arg3.1, and activation of Wnt signaling in amyloid‐accumulating Sgo1^−/+^. (a) The “amyloid‐β accumulation cycle” hypothesis (Rao et al., 2020). The “amyloid‐β accumulation cycle” hypothesis purports the occurrence of vicious cycles of events leading to amyloid‐β accumulation (see Introduction). Among a few mysteries in the hypothesis is the growth signaling driving the amyloid‐β accumulation cycle. (b) Key growth signaling pathways that are misregulated in human AD patients. AKT, AMPK, MAPK, and GSK3 are among the growth signaling misregulated in human AD and proposed to be involved in the disease process. GSK3 targets ARC/Arg3.1 and β‐catenin with ubiquitylation‐mediated proteolysis. (c) Phosphorylated GSK3 α and β (inactive forms) increased in Aβ‐accumulating Sgo1^−/+^. We tested components of the growth signaling in (b). Amounts of pGSK3α (S21) and pGSK3β (S9), inactive forms of GSK3, increased significantly in Aβ‐accumulating Sgo1^−/+^, while the total amount of GSK3 showed only a minor change. Consistently, ARC/Arg3.1 amount also significantly increased. pMAPK^42/44^, pAMPK, PCNA, and pTBK1 did not show significant change. (d) Nuclear accumulation of pGSK3α (S21) in Sgo1^−/+^. Consistent with immunoblots in (c) Aβ‐accumulating Sgo1^−/+^ showed accumulation of pGSK3α in the nucleus, both in the hippocampus and in the cortex. Age‐matched wild‐type showed no such pGSK3α accumulation. Enlarged panels for localization details. (e) Cytoplasmic accumulation of pGSK3β (S9) in Sgo1^−/+^. Aβ ‐accumulating Sgo1^−/+^ showed accumulation of pGSK3β in the cytoplasm, both in the hippocampus and in the cortex. Enlarged panels for localization details. (f) ARC/Arg3.1 was accumulated in the nucleo‐cytoplasm. ARC/Arg3.1 was accumulated in the nucleo‐cytoplasm, in both the hippocampus and the cortex, in Sgo1^−/+^. Enlarged panels for localization details. In wild‐type, IHC signals for ARC/Arg3.1 were much weaker, if any. (g) Another GSK3 target β‐catenin was enriched in the nuclei of Sgo1^−/+^, indicating Wnt signaling activation. Consistent with GSK3 inactivation, nuclear translocation of β‐catenin, a sign of canonical Wnt signaling activation and cell fate toward cell cycle and mitosis, was observed in Sgo1^−/+^ as distinct nucleo‐cytoplasmic signals in both the hippocampus and the cortex. β‐catenin IHC signals in wild‐type were weak, if any (enlarged panels)

Based on the “amyloid‐β accumulation cycle” hypothesis (Figure 5a), we predicted that one or more growth signaling pathways may be activated in amyloid‐β‐accumulating middle‐aged Sgo1^−/+^ brains. Several key growth signaling pathways that are misregulated in human AD patients (Figure 5b) were selected and investigated to determine whether they indicate aberrant activation at the middle age (12 months) to late middle age (18 months) transition in the Sgo1^−/+^ mice, at which time Aβ accumulation occurred.

Activated GSK3 leads proteasomal degradation of β‐catenin (canonical Wnt signaling) and degradation of ARC/Arg3.1 (Gozdz et al., 2017). ARC/Arg3.1 was increased in AD patients and was suggested to be a causal protein for AD development (Wu et al., 2011). Thus, we tested the GSK3‐ARC/Arg3.1 axis. We detected significant increases in (a) phosphorylated GSK3α (S21) and GSK3β (S9), inactive forms of GSK3α and β, and (b) ARC/Arg3.1 (Figure 5c–e). Consistent with GSK3 inactivation, we observed nuclear‐localizing β‐catenin (Figure 5f). ARC/Arg3.1 transcription is regulated by the MAPK/ERK pathway, as the MEK inhibitor PD098059 blocked activation of Arc/Arg3.1 transcription by forskolin (Waltereit et al., 2001). However, there was no significant difference in total amount of pMAPK42/44 and AKT downstream pAMPK in the Sgo1^−/+^ model mice, suggesting that the Arc/Arg3.1 increase may be mainly posttranslational, rather than transcriptional (Figure 5c).

We also tested activation of the cGAS‐STING pathway, which is involved in detection of cytosolic DNA and innate immunity against virus. The cGAS‐STING pathway can be activated with genomic instability and generation of fragmented DNA in cytoplasm (Bakhoum et al., 2018), and thus was suspected to be activated in the model. However, signs of significant activation of the cGAS‐STING pathway were not observed with the markers tested (i.e., pTBK1, cGAS, pSTING, and pIRF3) in Sgo1^−/+^ brains (not shown).

Our results suggest that accumulation of Aβ in middle‐/late middle‐aged Sgo1^−/+^ mice is driven by, at least in part, misregulation of the GSK3‐ARC/Arg3.1 axis and activation of Wnt signaling. This GSK3 inactivation is quite different from EOAD mouse models with hyper‐activated GSK3 (see Section 4).

### 3.6 Protein panel analysis

**FIGURE 6:**
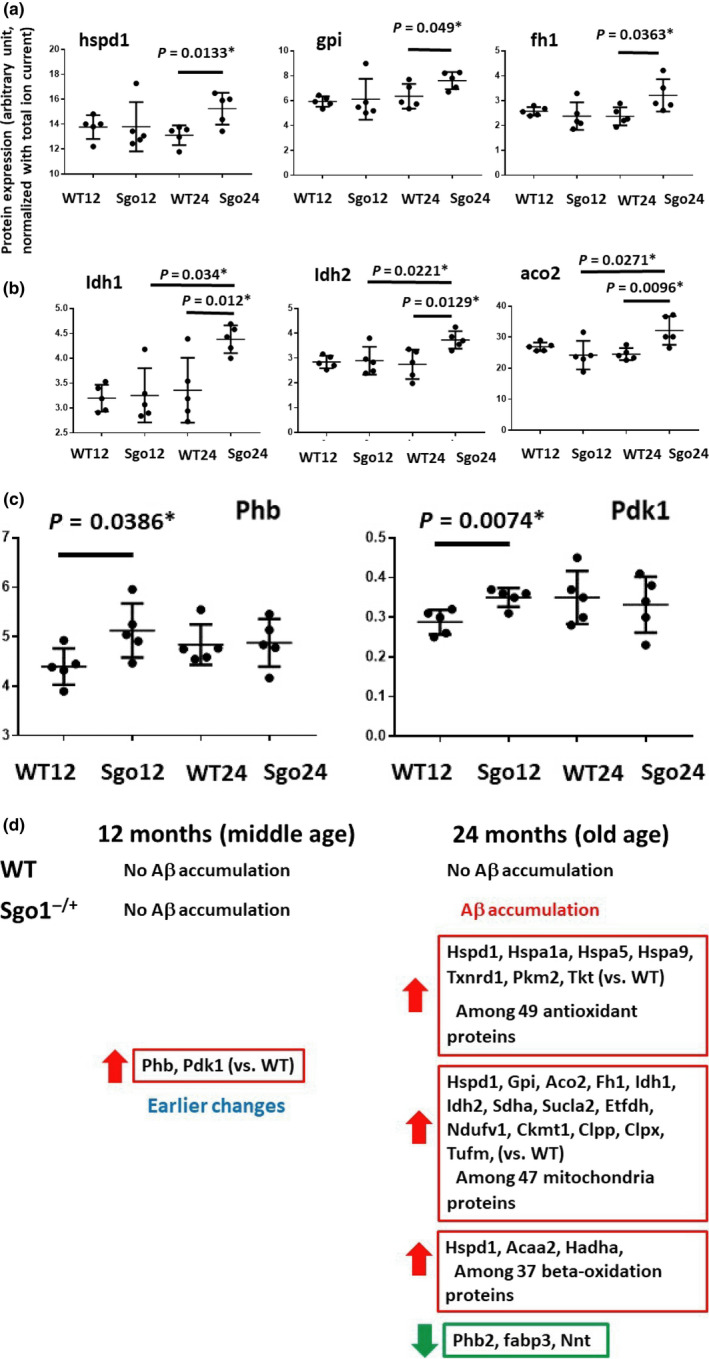
Protein misregulations in Sgo1^−/+^ in human AD‐relevant proteins (i.e., antioxidant proteins, mitochondrial energy metabolism proteins, and β‐oxidation and peroxisome proteins). (a) Examples of proteins indicating an Sgo1^−/+^‐specific increase in 24‐month‐old brains. Hspd1 (Heat Shock Protein Family D [Hsp60] Member 1) is a mitochondrial chaperone. Gpi (Glucose‐6‐phosphate isomerase) and Fh1 (Fumarate Hydratase) are involved in energy generation. (b) Examples of proteins indicating both an Sgo1^−/+^‐specific increase at 24 months and age‐dependent increase in Sgo1^−/+^ (12 months vs. 24 months). Idh1 (Isocitrate Dehydrogenase [NADP(+)] 1, Cytosolic), and Idh2 (Isocitrate Dehydrogenase [NADP(+)] 2, Mitochondrial) are involved in energy generation. Aco2 (aconitase 2, mitochondrial) localizes in mitochondria and is a part of the TCA cycle. (c) Prohibitin (Phb) and Phosphoinositide‐dependent protein kinase 1(Pdk1) uniquely indicated increases in middle‐aged (12‐month‐old) Sgo1^−/+^ mice, preceding amyloid‐β accumulation at 24 months. Phb and Pdk1 are also progressively decreased in human olfactory bulb‐AD proteomic analysis (see text). (d) The majority of misregulations were increases at old age, while Phb2 (Prohibitin2), Fabp3 (Fatty Acid‐binding Protein 3), and nnt (Nicotinamide Nucleotide Transhydrogenase) showed decreases. Many of these proteins are also misregulated in human AD (see text)

Next, we explored other aging‐associated factors as contributors to late‐onset amyloid‐β accumulation in Sgo1^−/+^ brains. The analyses included quantitative mass spectrometry‐based protein expression panels for 49 antioxidant proteins, 47 mitochondrial energy metabolism proteins, and 37 β‐oxidation and peroxisome proteins. Not counting overlapping proteins, the analysis quantified 130 proteins in four groups of mice (12‐month‐old wild‐type and Sgo1^−/+^ mice, 24‐month‐old wild‐type and Sgo1^−/+^ mice). Figure 6a shows examples of proteins indicating an Sgo1^−/+^‐specific increase in 24‐month‐old brains. Figure 6b shows examples of proteins indicating both an Sgo1^−/+^‐specific increase at 24 months and an age‐dependent increase in Sgo1^−/+^ brains (12 months vs. 24 months). Figure 6c shows expression profiling for Prohibitin (Phb) and Phosphoinositide‐dependent protein kinase 1(Pdk1), which indicated an earlier increase in Sgo1^−/+^ mice than in age‐matched controls. Figure 6d summarizes the results; the majority of misregulations were increases at 24 months of age in (aged) Sgo1^−/+^ brains. Among 130 proteins tested, 25 proteins were misregulated in Sgo1^−/+^ mice compared with wild‐type mice (more details in Figure S1).

Notably, increases in Phb and Pdk1 were identified in 12‐month‐old (middle‐aged) Sgo1^−/+^ mice. Progressive Phb and Pdk1 decreases were identified in olfactory bulb neuroproteomics for human patients with AD (Lachén‐Montes et al., 2017), indicating that the same pathway was impacted in Sgo1^−/+^ mice, albeit toward an apparently opposite direction. Additional proteins indicating misregulation in Sgo1^−/+^ mice were reported as misregulated in human AD‐omics analysis. For example, sporadic human AD patients showed a significant ~40%–60% increase in expression levels of select genes activated by the mtUPR, including mitochondrial chaperone hspd1 and mitochondrial protease clpp (Beck, Mufson, & Counts, 2016), which were recapitulated in Sgo1^−/+^ mice. Perredoxin6 (prdx6) increased with human AD, which may represent an oxidative stress defense mechanism (Power et al., 2008). Mitochondrial aconitase (aco2) expression in human AD with mild cognitive impairment was reported to be lower (Khodagholi, Shaerzadeh, & Montazeri, 2018; Mangialasche et al., 2015), while Sgo1^−/+^ mice showed a possible compensatory increase. Oxidative inactivation of Pyruvate kinase isozyme M2 (PKM2) was proposed to be involved in the progression of AD from mild cognitive impairment (Butterfield et al., 2006). NADH:Ubiquinone Oxidoreductase Core Subunit V1 (NDUFV1) was among the critical hippocampal genes and pathways that might be involved in the pathogenesis of human AD, identified via bioinformatics (Zhang et al., 2015). Fatty acid‐binding protein 3 (Fabp3) is a human AD biomarker in cerebrospinal fluid and in sera (Chiasserini et al., 2017; Höglund et al., 2017). A Phb2 decrease in Sgo1^−/+^ mice may additionally contribute to cohesinopathy, as depletion of Phb2 by RNA interference caused premature sister‐chromatid separation and mitotic arrest by spindle‐checkpoint activation, a near‐identical phenotype to that of the Sgo1 defect, indicating a functional similarity of Phb2 with Sgo1 during mitosis (Takata et al., 2007).

## 4 DISCUSSION

The present study identified 15–18 months of age (late middle age) as the age when spontaneous cerebral amyloid‐β accumulation became evident in the Sgo1^−/+^ model mice. In human LOAD, cerebral amyloid‐β also begins to accumulate in middle to late middle age, 15–20 years before the cognitive symptoms of AD manifest. Thus, the Sgo1^−/+^ model, carrying no mutations in APP or PSEN1, recapitulates the late‐onset and sporadic aspect of amyloid‐β accumulation. This identification of amyloid‐β accumulation timing helps to establish experimental conditions for using the model for testing an AD drug candidate, especially for disease intervention initiated in middle age. To which degree the internal pathology of Aβ accumulation affects the animals' cognition, behavior, and memory as external LOAD symptoms is a major research interest, which will be addressed in the future studies.

While we investigated potential causes for the late‐onset Aβ accumulation, the Sgo1^−/+^ model revealed two surprises that are different from conventional EOAD mouse models; (1) GSK3 activation status, and (2) β‐catenin and Wnt signaling behavior. GSK3 is thought to be involved in Aβ generation (Phiel, Wilson, Lee, & Klein, 2003). In the 5xFAD EOAD mouse model, both the GSK3α and β isoforms are hyperactive, and GSK3 inhibitor or GSK3α shRNA ameliorated senile plaque formation (Avrahami et al., 2013). In the PS1 APP EOAD mouse model, β‐catenin and p‐β‐catenin amounts are comparable to those in wild‐type controls at 6 months of age, yet at 18 months of age, PS1 APP mice displayed a significant decrease in β‐catenin. GSK‐3β inhibitory phosphorylation (S‐9) showed a marked decrease by 18 months in PS1 APP mice (Jimenez et al., 2011). These results in EOAD mice are consistent with an interpretation that GSK3 activation is a contributor of AD development, and GSK3 inhibition would be beneficial to manage AD.

However, the present study indicated age‐associated GSK3α/β inactivation that is concurrent with Wnt activation, ARC/Arg3.1 accumulation, and Aβ accumulation, in Sgo1^−/+^ model mice. One way to interpret the discrepancy may be in the context of existing Aβ amount. EOAD mice with already high expression of Aβ may increase GSK3 activity as a result of the Aβ increase, while GSK3 inactivation in middle‐aged Sgo1^−/+^ with a physiologically limited amount of Aβ plays dual roles of (a) activating Wnt signaling and (b) increasing ARC, both of which can increase Aβ. In other words, GSK3 may play different roles in early Aβ accumulation (represented in middle‐aged Sgo1^−/+^) and in later stages of AD pathology development with high levels of Aβ (represented in EOAD models). This possibility should be noted in planning for the use of GSK3 inhibitor for AD therapy (with advanced AD pathology) or for intervention (with limited AD pathology). Whether a notable decrease in GSK3 activity occurs in middle age at an early phase of human LOAD, or whether GSK3 inhibitor increases amyloid‐β in middle‐aged wild‐type mice, remains to be tested. GSK3 inhibitor lithium salts are clinically used for bipolar disorder. Patients with bipolar disorder are at higher risk for developing AD (Rochoy, Bordet, Gautier, & Chazard, 2019), suggesting the risk of GSK3 inhibition leading to AD.

Among various neuroinflammatory proteins proposed to be involved in human AD (see Figure 3a), INF‐λ and TNF‐α were co‐expressed in brains of Sgo1^−/+^ mice in the present study. Several reports suggest the involvement of INF‐λ and TNF‐α in human AD pathology development. INF‐λ and TNF‐α levels were higher, as was nitric oxide production, in AD patients in mild and severe stages compared with patients in earlier phases (moderate stage and mild cognitive impairment), indicating progressive increases in INF‐λ and TNF‐α in human AD patients (Belkhelfa et al., 2014). Bhaskar et al. (2014) showed that activated TNF‐α and the c‐Jun Kinase (JNK) signaling pathway led neuronal cells to cell cycle progression toward the mitotic cycle, which was followed by neuronal cell death. This sequence of events is consistent with the aforementioned “amyloid‐β accumulation cycle” hypothesis (Rao et al., 2020). Mouse primary astrocytes treated with both INF‐λ and TNF‐α displayed significantly increased levels of astrocytic APP, BACE1 (an APP‐Aβ conversion enzyme), and secreted Aβ40, suggesting a role of INF‐λ and TNF‐α as priming factors for astrocytes to produce amyloid‐β (Zhao, O'Connor, & Vassar, 2011). From the present study, targeting INF‐λ and/or TNF‐α, and assessing the effects on amyloid‐β accumulation, has emerged as a new approach of interest.

Another set of results, indicating protein misregulations in Sgo1^−/+^ and human AD in common pathways, also suggests the utility of Sgo1^−/+^ mice as a study model for LOAD development. A critical point in interpreting the protein panel data is whether the misregulation is causal to AD, or is compensatory/antagonizing to AD development. As 24‐month‐old Sgo1^−/+^ mice show amyloid‐β accumulation colocalizing with p‐TAU, but not extensive neurofibrillary tangles or neurodegeneration (Rao, Farooqui, Asch, et al., 2018; Rao, Farooqui, Zhang, et al., 2018), we suspect that the Sgo1^−/+^ mouse model represents a relatively early phase in LOAD development, and speculate that many of the misregulations are compensatory. Itemized tests and validation will be needed.

If the notion that the Sgo1^−/+^ model represents an early phase of LOAD is correct, the model may be useful in two ways: (a) to test AD intervention drug candidates and assess them with amyloid‐β reduction, and (b) to challenge the mouse with AD facilitator candidates and validate the “facilitator” with assessments of whether advanced AD pathology (i.e., Aβ plaques and neurofibrillary tangles [NFT]) would develop. Alternatively, if the mild plaque/NFT pathology in current Sgo1^−/+^ model is due to the differences in human and mouse protein structures, developing a “humanized” Sgo1^−/+^ model may yield a model that indeed recapitulates “plaques and tangles.” Such a model would be a highly LOAD‐relevant research tool.

Elucidating the mechanism by which amyloid‐β starts accumulating in brains in non‐symptomatic early phases would reveal effective interventions for LOAD. In the present study, we observed accumulation of amyloid‐β and its co‐localization with neuroinflammatory markers, specifically in the aged Sgo1^−/+^ model mice and not in age‐matched control mice. The results suggest new possible scenarios occurring at an early stage of amyloid‐β accumulation; (a) amyloid‐β accumulation during prolonged mitosis (Rao et al., 2020; Rao, Farooqui, Zhang, et al., 2018) triggers inflammation markers, possibly as a part of mitotic catastrophe; and, alternatively, (b) cells with accumulated inflammation markers go through prolonged mitosis and accumulate amyloid‐β (Rao et al., 2020; Rao, Farooqui, Zhang, et al., 2018).

Overall, our present findings (a) caution that the use of GSK3 inhibitors in middle age may be a potential facilitator of amyloid‐β accumulation, (b) further add to the body of knowledge of the Sgo1^−/+^ model's similarities to human AD, suggesting its potential as an animal model of spontaneous amyloid‐β accumulation and LOAD, and (c) support the model to claim a unique niche among existing and prospective AD research models.

## CONFLICTS OF INTEREST

The authors declare no conflicts of interest.

## AUTHORS' CONTRIBUTIONS

H.Y. Yamada contributed to all aspects of the project. M. Farooqui and A. Madhavaram contributed key data generation. Y. Zhang contributed animal maintenance and sample collection. C.V. Rao and A.S. Asch provided material support and intellectual input.

## ETHICAL APPROVAL

Not applicable for human subjects. Animal usage has been reviewed and approved by the OUHSC IACUC.

## Supporting information

Fig S1

## Data Availability

Raw data are available upon request from the corresponding author(s). Material transfer is subjected to negotiation with the PI and OUHSC. A certain use of Sgo1^−/+^ mouse is protected by intellectual property rights of the University of Oklahoma.
