# Supplementary figures and images for: GSK3‐ARC/Arg3.1 and GSK3‐Wnt signaling axes trigger amyloid‐β accumulation and neuroinflammation in middle‐aged Shugoshin 1 mice
Source: Aging Cell. 2020 Aug 28;19(10):e13221. doi: 10.1111/acel.13221 (PMC7576275; doi:10.1111/acel.13221)

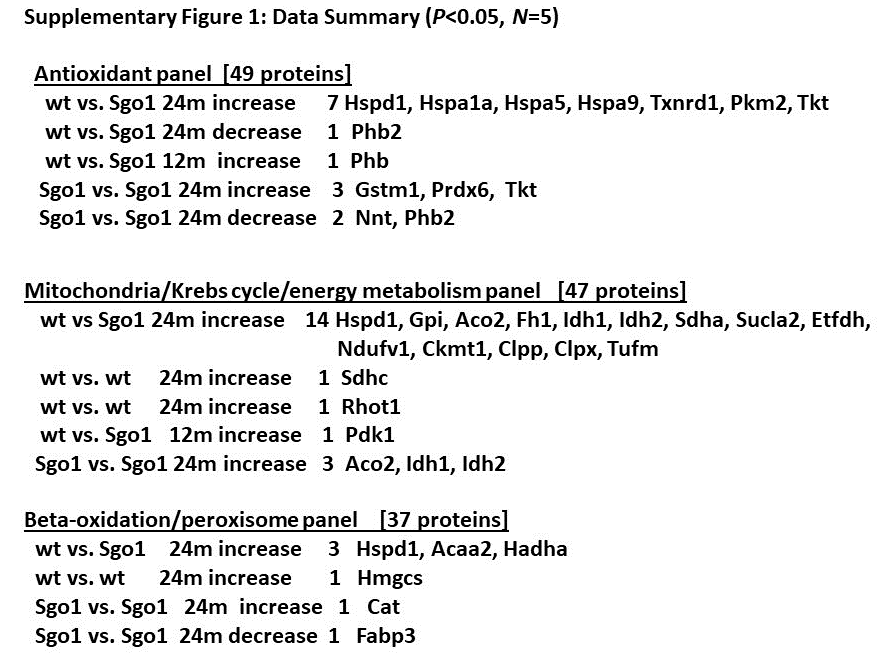

Supplement: Supplementary file 1 — Fig S1 [file ACEL-19-e13221-s001.tif]
